# Editorial: In Search of *In vivo* MSC

**DOI:** 10.3389/fcell.2017.00060

**Published:** 2017-05-29

**Authors:** Simone Pacini, Mario Petrini

**Affiliations:** Department of Clinical and Experimental Medicine, University of PisaPisa, Italy

**Keywords:** *in vivo* MSC, MSCs, mesenchymal stem cells, multipotent stem cells, multipotent cell differentiation, bone marrow transplantation, adult stem cells

The concept of multipotent mesenchymal stromal cells (MSCs) arose from the work of Friedenstein et al., in which the authors observed that culturing human bone marrow (hBM) cell suspensions, lead to isolation of proliferating adhered colonies of fribroblastoid cells able to differentiate into chondrocytes or osteoblasts, *in vitro* (Friedenstein et al., 1968), and *in vivo* (Friedenstein et al., 1974). Emerging interest in identifying the MSCs *in vivo* counterpart lead to increasing number *of ex vivo* isolating procedures. Nonetheless, any effort failed to describe a definitive accepted protocol, and significantly contributed to the ongoing confusion in the *in vivo* MSC identity (Trombi et al., 2009; Cordeiro-Spinetti et al., 2014; Montali et al., 2016). This resulted in the perception that these *in vivo* progenitors were highly heterogeneous cell population.

Due to this uncertainty, after several decades from its first description the colony-forming units assay remain the elective indirect method to quantify mesenchymal progenitors in a cell suspension. Nonetheless, it is known that colonies are highly heterogeneous and arise from a single progenitor with different level of “stemness” or “commitment” (Muraglia et al., 2000; Russell et al., 2010), which may correlate with colony size and cell distribution (Kuznetsov et al., 2009). In this issue, Eric Cordeiro-Spinetti and colleagues discuss how variability in FBS could affect CFU-F size, frequency and morphology. Authors suggest a “*Selective Growth Hypothesis*” where distinct mesenchymal progenitors could be differentially induced to proliferate or to remain quiescent depending on serum concentration of different growth factors (Cordeiro-Spinetti et al., 2014).

We had also proposed the “*Selective Growth Hypothesis*” explaining the emergence of a new stromal progenitor in primary cultures of hBM cells (Trombi et al., 2009). These cells, recently renamed “Mesangiongenic Progenitor Cells” (MPCs) retaining both mesengenic and angiogenic potential (Montali et al., 2016). MPCs could be easily obtained culturing hBM cells in minimal essential medium supplemented with pooled human AB type sera (PhABS) in place of FBS. However, the efficient MPC isolation had always been affected by a great lot-to-lot variability. Here, Montali et al. demonstrated that this variability is in close correlation with the concentration of few specific growth factors, supporting the hypothesis propose by Cordeiro-Spinetti's group.

Most of the controversies still debated on MSC differentiating/supporting potential, could be ascribed to the heterogeneity of MSC culture products and the variability related to their fabrication. Unfortunately, there are still no phenotypical and molecular markers that could be efficiently applied evaluating the potential of a MSC primary culture. Here Wilson et al. investigated the possibility to apply glycan profiling to predict the cell differentiating potential. In their interesting new approach, Authors compare *N*-Glycomes from different MSC clones showing distinct osteogenic activity, *in vitro*.

In addition, one the most debated controversy in MSC biology is represented by the genuine angiogenic potential of these cells. In this issue, Iacopo Petrini and I try to answer to the question: “Are MSC angiogenic cells?” hypothesizing that the presence of undetected MPCs, could be responsible for the angiogenic potential of MSC cultures reported by some Authors but not yet definitely demonstrated (Pacini and Petrini).

Here above, the “*Selective Growth Hypothesis*” has been discussed in correlation to the concentration of particular growth factors. Nonetheless, many others culture parameters could deterministically and stochastically varying during the culture time as cell density, pH, temperature, nutrient impoverishment, medium evaporation and oxygen tension (Pacini, 2014), resulting in cell stressing conditions. Under those conditions some particularly stress resistant sub-populations could be positively selected. Ciavarella et al. explored this hypothesis, applying extremely adverse conditions to cultures of vascular wall-derived MSC and reporting interesting results on cell survival. Moreover, Antonini et al. suggest that also nanotopography of the culture surface should be taken in account as further culture determinant Antonini et al. These results corroborate the hypothesis that an untreatable number of external factors contributing to the heterogeneity of *in vitro* isolated/expanded populations.

BM still represents the most extensively studied source of MSCs and the hypothesis that these cells could migrate from BM to other injured organs triggering tissue regeneration has gaining evidence. Kimura et al. demonstrate the recruitment of BM-derived MSC progenitors in periodontal tissue defects produced in a chimeric mouse model. Moreover, it has been demonstrated that these cells could be recruited also by tumor tissues in respond to inflammatory molecules and MSCs have been considered contributing to cancer progression. In this topic, Kudo-Saito summarizes current knowledge about the role of MSCs in tumor aggravation.

BM-derived cells could also be applied to allogeneic BM transplantation (BMT) where hemopoietic stem cells (HSCs) and MSCs could be systemically administered, restoring normal hemopoiesis thank to their “*homing*” ability and BMT also represents a therapeutic approach to autoimmune diseases. However, some reports demonstrated that stroma cells could be trapped in the liver when are systemically infused. To overcome this obstacle, Professor Ikehara's group defined the methodology to directly inject BM into the bone cavity, demonstrating that this methods results more effective in allogenic BMT. This Research Topic hosts an interesting review article about the application of *intra-bone marrow-bone marrow transplantation* (IBM-BMT) in the treatment of rheumatoid arthritis and malignant tumors (Li et al.).

Expanded and *ex vivo* isolated MSCs from different tissues of origin has been under extensive investigations also for the treatment of heart diseases, which have a great impact on public health. Professor Paolo Madeddu, in collaboration with Paolo Caputo and Elisa Avolio, here presented a comprehensive review on stem cell therapies for congenital heart diseases (Avolio et al.).

Although MSCs might be considered the most intensely studied adult multipotent cells, comparison of existing pre-clinical and clinical data reveals a significant level of uncertainty. Most of the reduced predictability of pre-clinical studies could be ascribed to the uncertainty about the genuine MSC *ex vivo* ancestors. The identification of these cells could be affected by an untreatable number of variables related to the donors, the tissue of origins and cell manipulations.

Thus, the application of highly purified cell populations, finely characterized and unequivocally defined by specific manipulation procedures is of primary importance. All the contributions collected in this Research Topic strongly suggest avoiding the generic definition of MSC to characterize the various multipotent cell populations object of study.

## Author contributions

SP: Revised the literature and drafted the article, MP: Revised the literature and final approve.

### Conflict of interest statement

The authors declare that the research was conducted in the absence of any commercial or financial relationships that could be construed as a potential conflict of interest.
